# Prognostic Value of Preoperative Serological Biomarkers in Patients Undergoing Curative-Intent Cytoreductive Surgery for Colorectal Cancer Peritoneal Metastases

**DOI:** 10.1245/s10434-022-12736-1

**Published:** 2022-11-09

**Authors:** Antoine El Asmar, Martin Delcourt, Leonel Kamden, Charif Khaled, Ali Bohlok, Michel Moreau, Francesco Sclafani, Vincent Donckier, Gabriel Liberale

**Affiliations:** 1 Department of Surgical Oncology, Institut Jules Bordet, Université Libre de Bruxelles (ULB), Brussels, Belgium; 2 Faculty of Medicine, Université Libre de Bruxelles (ULB), Brussels, Belgium; 3 Department of Biostatistics, Institut Jules Bordet, Université Libre de Bruxelles (ULB), Brussels, Belgium

## Abstract

**Background:**

Cytoreductive surgery (CRS) for peritoneal metastases of colorectal cancer (PMCRC) is associated with a high risk of postoperative morbidity, thus making patient selection of upmost importance. Further to data showing an association between preoperative serological biomarkers and patient outcome in various solid tumors, in this study we aim to evaluate their prognostic value in patients with PMCRC treated with curative intent.

**Patients and Methods:**

This is a retrospective study including patients with PMCRC treated by complete CRS ± HIPEC at our institution between 2011 and 2020. Preoperative serological biomarkers, along with other standard clinicopathological variables, were studied to determine their prognostic value.

**Results:**

A total of 94 out of 108 patients met the inclusion criteria. Forty-three patients (46%) presented with synchronous PM. The median peritoneal cancer index (PCI) was 6. On univariate analysis, a higher neutrophil-to-lymphocyte ratio (NLR) was associated with poor prognosis in terms of overall survival (OS) [cutoff 3.567, hazard ratio (HR) 2.8 (1.4–5.3), *p* = 0.002], whereas a higher platelet-to-lymphocyte ratio (PLR) predicted favorable prognosis in terms of disease-free survival (DFS) [cutoff 185.4, HR 1.9 (1.07–3.53), *p* = 0.030]. On multivariate analysis, NLR > 3.567, positive lymph nodes (LNs), and PCI > 7 were independent predictive factors for worse OS, whereas NLR > 3.567 and positive LNs were significantly associated with worse DFS. PLR > 185.4 was associated with better DFS.

**Conclusion:**

High preoperative NLR (> 3.567) and PLR (> 185.4) can predict outcome of patients with PMCRC treated by complete CRS ± HIPEC.

Colorectal cancer represents the second leading cause of mortality worldwide. The peritoneum represents the second most common metastatic site after the liver, with around 13% of patients developing peritoneal metastases (PM), either synchronously or metachronously.^1^ PM are generally associated with an unfavorable prognosis; patients with synchronous, peritoneum-only metastasis have an overall survival (OS) of approximately 8 months.^2,3^ However, in selected patients, cytoreductive surgery (CRS), with or without hyperthermic intraperitoneal chemotherapy (HIPEC), is an established treatment option with 5-year overall survival (OS) rates of 30% and 38% in tumors of colonic and rectal origin, respectively.^4^ Recently, the PRODIGE 7 study has reported the lack of benefit from adding HIPEC to CRS in PMCRC patients treated with 6 months of perioperative systemic chemotherapy.^5^ However, the role of HIPEC in patients treated with upfront CRS without systemic chemotherapy remains unknown.^6^

CRS is associated with a postoperative Clavien–Dindo grade (CDG) 3–4, morbidity rate of 31%.^7^ This highlights the importance of selecting those patients who are most likely to benefit from such procedure.^4^ The most important prognostic factors for PMCRC remain the peritoneal cancer index (PCI) and the radicality of surgery,^8^ and major efforts have been made to refine patient selection based on different PCI thresholds and to improve intraoperative detection and treatment of PM.^9,10^ However, patient selection in this setting is still suboptimal, and new prognostic factors are needed to implement individualized treatment strategies with an optimal risk–benefit ratio.

Serological biomarkers such as CRP and albumin (alb) have been studied in multiple solid tumors as predictive factors for postoperative and oncological outcomes.^11–14^ In addition, the prognostic value of other inflammatory biomarkers such as neutrophils (N), lymphocytes (L), platelets (P), and monocytes (M) and their ratios (NLR, PLR, MLR) have increasingly been investigated, in both the preoperative and the palliative setting of many solid tumors.^15,16^ These ratios were mostly studied in patients with primary CRC, with or without synchronous PM,^16,17^ or postoperatively in patients with PMCRC,^18^ but limited data are available on their value as prognostic factors before CRS for PMCRC.^19^

The aim of our study is to evaluate the prognostic value of a number of preoperative biomarkers in patients undergoing curative CRS ± HIPEC for PMCRC.

## Patients and Methods

### Study Design and Inclusion Criteria

This is a retrospective, single-center study. Eligibility was limited to patients who underwent CRS ± HIPEC for PMCRC with curative intent (complete macroscopic resection, CC-0, or R0/1) at our institution between June 2011 and June 2020. Patients who had a palliative intervention were excluded, as well as those who suffered from an acute inflammatory event (such as bowel occlusion or intra-abdominal or systemic infection) or who had received neoadjuvant chemotherapy (NAC) within 30 days prior to surgery to avoid any influence on the biochemical markers.

During the study period, our policy for treating PMCRC was to perform explorative laparoscopy to evaluate the PCI and propose CRS plus HIPEC (intraperitoneal oxaliplatin 460 mg/m^2^, and systemic 5-fluorouracil), to patients with limited disease (PCI ≤ 16), whereas NAC followed by CRS plus HIPEC +/− pseudoadjuvant chemotherapy (depending on the degree of tumor regression on the surgical specimens) was administered to patients with extensive disease (PCI > 16), or if major resections were required owing to vascular invasion or widespread small bowel involvement.

### Data Collection

Patient data, including demographic, clinical, pre/intraoperative, biochemical, and histopathological data were collected using our prospectively maintained database and institutional electronic medical records system (Oribase). Biochemical markers were extracted from preoperative blood samples. The study was approved by the Institut Jules Bordet Ethics committee (CE3414). Variables included were age, gender, BMI, primary tumor characteristics (histology, lymph nodes status, grade, location, MSI, RAS), timing of the PM, PCI, neoadjuvant treatments, and whether or not HIPEC was performed. Serological biomarkers were collected: CEA, Ca19-9, CRP, albumin, neutrophils (N), lymphocytes (L), platelets (P), and monocytes (M); and their ratios were calculated: CRP-to-albumin ratio, neutrophil-to-lymphocyte ratio (NLR), platelet-to-lymphocyte ratio (PLR), and monocyte-to-lymphocyte ratio (MLR).

### Statistical Analysis

Statistical analyses included a descriptive analysis of the study population and its characteristics expressed as mean (+/− standard deviation) and proportion (%) depending on whether the variables were continuous or discrete. The association between the included variables and overall survival (OS) and disease-free survival (DFS) was studied using the Kaplan–Meier (KM) test and the Cox regression model. Time to event was defined as

the delay between the date of diagnosis of carcinosis and death (OS) and the date of the subsequent event which occurs first, recurrence or death (DFS). The cutoffs for the studied ratios were defined using the 25th, 50th, and 75th percentiles (p25, p50, and p75) to determine a certain value above or below which this ratio predicts the prognosis of patients. For the other predictors, clinically significant or internationally recognized cutoffs were used. Multivariate Cox regression analysis was performed to adjust for potential confounding. Covariates initially introduced into the multivariate models were the statistically significant variables/ratios, and those with borderline significance (*p* < 0.1) on univariate analysis. Selection of the predictors relied on a backward procedure. Statistics were conducted using SAS version 9.4.

## Results

### Demographics

During the study period, 108 patients presenting with PMCRC underwent CRS ± HIPEC. Out of those, 14 were excluded, 10 owing to incomplete (R2a) resection (the intestinal mesentery harbored hundreds of small nodules that were subjected to electrofulguration but not resection) and 4 owing to loss to follow-up straight after surgery. Table 1 summarizes the baseline characteristics of the 94 eligible patients. There was a predominance of females (63%), with the majority having an intermediate grade adenocarcinoma and the primary tumor located in the right colon (52% each). PMCRC were metachronous (i.e., diagnosed > 6 months after resection of the primary tumor) in 47.9% of patients, and the median PCI was 6. HIPEC was administered in 88.3% of patients.

**Table 1:** Demographic, clinical, and histopathological characteristics of patients presenting with PMCRC

Variable	Patients	Percentage
*Gender*		
Male	35	37.2%
Female	59	62.8%
Age, median (years)	59	
BMI, median (kg/m^2^)	24.7	
*Primary tumor histology*		
Adenocarcinoma	68	72.3%
Mucinous adenocarcinoma	26	27.7%
*Primary tumor grade*		
Low	27	28.7%
Intermediate	49	52.1%
High	18	19.2%
*Primary tumor location*		
Right colon	41	43.6%
Transverse colon	6	6.4%
Left colon/sigmoid	35	37.3%
Rectum	10	10.6%
Small intestine	2	2.1%
*Microsatellite instability (MSI)*		
Yes	7	13.3%
No	50	87.7%
*RAS mutation*		
Yes	35	50.7%
No	34	49.3%
*Timing of peritoneal metastases*		
Metachronous	45	47.9%
Synchronous	49	52.1%
PCI, median	6	[3–10]*
*Neoadjuvant treatment before CRS*		
No	67	71.3%
Yes	27	28.7%
*HIPEC*		
Yes	83	88.3%
No	11	11.7%
CEA, median (pre-CRS, µg/L)	4.4	[0.5–407]**
CA19-9, median (pre-CRS, U/mL)	17.1	[0.6–3490]**

*IQR interquartile range

**Interval: [min–max]

*CRS* cytoreductive surgery, *PCI* peritoneal carcinomatosis index

Median DFS and OS of the study population was 14 and 53 months, respectively, with a 5-year OS of 42%.

### Clinicopathological Variables

Among all clinicopathological factors analyzed, only N stage at the diagnosis of the primary tumor and PCI were prognostic (Table 2). Patients with positive lymph nodes (LNs) had a worse prognosis compared with those with negative LNs, with median OS of 45.5 versus 94.5 months, respectively (*p* < 0.01,) and median DFS of 16.2 versus 44.5 months, respectively (*p* = 0.001) (Table 2). Patients with PCI ≥ 7 had worse OS (45.5 months) compared with those with PCI < 7 (71.6 months, *p* < 0.05), while no difference in DFS was observed (Table 2). On multivariate analysis, the association between N stage of the primary tumor, DFS, and OS remained significant, while PCI was an independent predictive factor for OS (Table 4).

**Table 2:** Association of clinical, histologic, and serological variables with DFS and OS in patients undergoing CRS ± HIPEC for PMCRC (univariate analysis)

Variables	Median value	OS		DFS	
		Median OS (months)	*p*-value	Median DFS (months)	*p*-value
Lymph nodes	Negative	94.5	**0.0008**	44.5	**0.001**
	Positive	45.5		16.2	
PCI	< 7	71.6	**0.016**	17.9	0.07
	≥ 7	45.5		17.8	
BMI (kg/m^2^)	< 30	56.8	0.59	17.4	0.77
	≥ 30	53.9		23.9	
Primary tumor localization	Left colon	55.5	0.63	19.7	0.28
	Right colon	53.9		17.4	
Albumin (g/L)	< 43	52.3	0.19	12.1	0.10
	≥ 43	55.5		19.7	
CRP (mg/L)*	< 3.5	55.1	0.36	19.2	0.64
	≥ 3.5	52.4		15.2	
Ca19-9 (U/mL)*	< 39	44.5	0.86	18.4	0.70
	≥ 39	52.8		15.0	
CEA (µg/L)*	< 4.4	53.9	0.41	19.7	0.58
	≥ 4.4	52.8		16.7	
Neutrophils (×10 s^3^/µL)	< 4	55.5	0.44	19.2	0.14
	≥ 4	48.5		16.5	
Lymphocytes (×10 s^3^/µL)	< 1.6	55	0.11	19.7	0.10
	≥ 1.6	50.5		16.5	
Monocytes (×10 s^3^/µL)	< 0.53	56	0.20	19.3	0.64
	≥ 0.53	47		16.5	
Platelets (×10 s^3^/µL)	< 240	53	0.98	17.1	0.80
	≥ 240	52		17	

Clinically significant *p*-values (< 0.05) are highlighted in bold

*Normal upper limit

### Lymphocytes, Monocytes, Neutrophils, Platelets, and CRP/Albumin Ratio

The individual values of lymphocytes, monocytes, neutrophils, platelets, CRP, albumin, Ca19.9, and CEA did not reveal any clinically significant association with either DFS or OS. Similarly, no association was found between CRP/Alb and survival outcomes.

### Neutrophil-to-Lymphocyte Ratio

A statistically significant association between NLR and OS was observed. Patients with NLR values ≥ 75th percentile (p75, 3.567) had median OS of 26.4 months versus 55.5 months for patients with NLR < 3.567 (HR 2.8, 95% CI 1.4–5.3, *p* < 0.01), but no significance was seen for DFS (Table 3). However, on multivariate analysis, NLR was confirmed to be an independent predictive factor for both DFS (HR 2.4, 95% CI 1.3–4.4, *p* < 0.01) and OS (HR 5.6, 95% CI 2.6–11.7, *p* < 0.01), as presented in Table 4.

**Table 3:** NLR and PLR correlation with OS and DFS (in months) on univariate analysis using the Cox model and patients’ survival in months

Ratio		OS			DFS		
		Median survival (months)	Hazard ratio (95% CI)	*p*	Median survival (months)	Hazard ratio (95% CI)	*p*
NLR	≤ 3.567	55.5	1	**0.002**	17.9	1	0.14
	> 3.567	26.4	2.8 (1.4–5.3)		11.0	1.5 (0.9–2.6)	
PLR	≤ 185.4	47.4	1.9 (0.96–3.9)	0.06	16.5	1.9 (1.07–3.53)	**0.030**
	> 185.4	87.2	1		43.0	1	

Clinically significant *p*-values (< 0.05) are highlighted in bold

**Table 4:** OS and DFS on multivariate analysis (Cox model)

Significant variables	OS		DFS	
	Hazard ratio (95% CI)	*p*-value	Hazard ratio (95% CI)	*p*-value
NLR > 3.567	5.6 (2.6–11.7)	**< 0.0001**	2.4 (1.3–4.4)	**0.004**
PLR ≤ 185.4*	–	–	2.0 (1.04–3.8)	**0.04**
Positive lymph nodes	4.4 (1.9–10.0)	**0.0005**	3.4 (1.8–6.7)	**0.0003**
PCI ≥ 7*	2.5 (1.4–4.7)	**0.002**	–	–

Clinically significant *p*-values (< 0.05) are highlighted in bold

*NLR, PLR, lymph nodes, and PCI were initially included in the multivariate analysis. PLR and PCI disappeared from the models after execution of the backward procedure for OS and DFS, respectively

### Platelet-to-Lymphocyte Ratio

A statistically significant association between PLR and DFS was observed (Table 3). Patients with PLR values ≥ 75th percentile (p75, 185.4) had median DFS of 43 months versus 16.5 months for patients with PLR ≤ 185.4 (HR 1.9, 95% CI 1.07–3.53, *p* = 0.03). The statistically significant independence of PLR as a predictive factor for DFS (HR 2.0, 95% CI 1.04–3.8, *p* < 0.05) was confirmed on multivariate analysis (Table 4).

## Discussion

The role of serological biomarkers in predicting prognosis of patients with PMCRC is still largely unknown. In this study, we found that, in addition to more conventional clinical variables such as N stage of the primary tumor and PCI, NLR and PLR were independent prognostic factors in this setting. On the other hand, all the other investigated biomarkers, including MLR and CRP/Alb, failed to predict prognosis.

Several studies have investigated the interaction between tumoral cells and the immune system by analyzing biochemical and hematological markers involved in this process.^22,23^ Lymphocytes are known for their antitumoral role, infiltrating the tumor area, attacking neoplastic cells, and ultimately mediating response to anticancer agents, especially immunotherapy. Neutrophils, on the other hand, can inhibit an adequate antitumoral response by suppressing the activity of lymphocytes, activated T-cells, and NK cells.^24^ Therefore, a high NLR may reflect an immune state of decreased antitumoral activity, and increased tumoral stimulation via neutrophil- and macrophage-derived cytokines, interleukins, and tumor growth factors,^15^ while a low NLR may be associated with an increased tumor immune surveillance, possibly mediated by tumor infiltrating lymphocytes (TILs). TILs are effective in delaying tumor progression by directly killing tumoral cells (CD8^+^ cytotoxic T lymphocytes), or by facilitating their detection and destruction (T helper lymphocytes, types 1 and 2), and have been shown to be predictive of good outcomes in many cancers.^25^

Many studies have highlighted the prognostic value of serological biomarkers in solid tumors; very few, however, have investigated their clinical utility in patients with peritoneal metastases, particularly of CRC origin. Rangarajan et al. revealed that a higher NLR was associated with poorer DFS and OS in patients with pseudomyxoma peritonei (PMP) of appendiceal origin, whereas Zager et al. showed that LMR had a negative prognostic significance in patients with PMCRC.^19,26^

In our study, positive lymph nodes after primary tumor resection and a high PCI were associated with worse prognosis, in line with previous series.^27^ In addition to these conventional parameters, NLR appeared as a strong, independent prognostic factor in our patient population, with median DFS and OS of 15 and 55.5 months, respectively, for patients with a ratio ≤ 3.567, almost twice as high as for those with a ratio > 3.567 (9.8 and 26.4 months, respectively). This finding is again consistent with the available data from the literature and supported by the differential role of neutrophils and lymphocytes in relation to the host immune response discussed above.

On the other hand, we observed an unexpected association between PLR and DFS. While patients with high PLR have been reported to have poor outcomes, in our study population a higher PLR (> 185.4) predicted better DFS, this also being confirmed after multivariate analyses. Interpreting such a finding is quite difficult as high platelet counts and low lymphocytic counts are generally markers of dismal prognosis for cancer patients.^25^ While it is possible that this could be just a false positive result, additional studies are warranted to further investigate the actual value of this parameter.

We appreciate that our study has several limitations, including, among others, the retrospective design, the small sample size, the analysis of patients from a single center (which might reduce the generalizability of our findings to other similar populations), and the lack of a statistical correction for the multiple testing (which might have increased the risk of random findings and false associations, such as for PLR). Nevertheless, it represents a step forward in the understanding of the clinical utility of serological markers that, in contrast to other tumors and settings, have long been neglected in patients with PMCRC. Further investigation of these in larger series and ideally prospective studies is warranted.

## Conclusions

In this study, preoperative NLR and PLR were independent prognostic factors in patients treated with complete CRS ± HIPEC for PMCRC. If validated in other studies, these serological markers, along with conventional clinicopathological variables and other biomarkers, could refine risk stratification in this setting, and potentially be used to optimize decision-making and implement patient-tailored treatment approaches.
